# Revision of the South American window fly genus *Heteromphrale* Kröber, 1937 (Diptera, Scenopinidae)

**DOI:** 10.3897/zookeys.84.774

**Published:** 2011-03-01

**Authors:** Shaun L. Winterton, Stephen D. Gaimari

**Affiliations:** California State Collection of Arthropods, Plant Pest Diagnostics Center, California Department of Food & Agriculture, 3294 Meadowview Road, Sacramento, California, USA

**Keywords:** *Heteromphrale*, Therevoid clade, Asiloidea, Scenopinidae

## Abstract

The Neotropical window fly genus Heteromphrale Kröber, 1937 is revised. Two previously described species (Heteromphrale chilensis (Kröber, 1937) and Heteromphrale cyanops (Edwards, 1932)) are redescribed while a new species (Heteromphrale blanca **sp. n.**) is described from Argentina. The male of Heteromphrale chilensis and female of Heteromphrale cyanops are described and figured for the first time, and a key to species is presented.

## Introduction

Window flies (Diptera: Scenopinidae) are a small family (*ca.* 420 species in 24 extant genera) of cosmopolitan asiloid flies with an adult body size rarely exceeding 5.0 mm. Scenopinids are distributed throughout all major biogeographical regions, but with significant continental endemism at the genus level (Kelsey 1973).

Heteromphrale Kröber, 1937 is one of several genera of Scenopinidae found in the Neotropics, a region also including Brevitrichia Hardy, 1944 (also Nearctic), Irwiniana Kelsey, 1971, Jackhallia Nagatomi & Liu, in Nagatomi et al. 1994, Pseudatrichia Osten Sacken, 1877 (also Nearctic), Metatrichia Coquillett, 1900 (cosmopolitan), and Scenopinus Latreille, 1802 (cosmopolitan). Heteromphrale was erected by Kröber (1937) to accommodate his previously described species Pseudatrichia chilensis Kröber, 1928 from Chile, with Pseudatrichia being a highly distinctive genus to which this species clearly does not belong. In his monographic revision of world Scenopinidae, Kelsey (1969) subsequently transferred Pseudomphrale cyanops Edwards, 1932 to Heteromphrale as the second species in the genus. These two previously described species of Heteromphrale were each known only from a single sex (i.e. the female of Heteromphrale chilensis and male of Heteromphrale cyanops). As a result of extensive collecting by Dr Michael Irwin in Chile and Argentina, the opposing sexes of both species are now available, along with males and females of a new species (Heteromphrale blanca sp. n.) described herein. Heteromphrale is revised with all species diagnosed and figured, and a dichotomous key to species presented. The key to genera in Woodley (2009) can be used to identify specimens to this genus, although the female of one species has a weakly emarginate posterior edge of sternite 8 (but not forming distinct posterolateral lobes as in Brevitrichia), and only one species has bulbous male epandrial lobes. As in recent papers using cybertaxonomic methods such as hypertext mark-up links to internet resources (e.g. online image databases, name registration in Zoobank, etc.) (Pyle et al. 2008; Winterton 2009), we have also extensively used such resources throughout the text.

## Materials and methods

Genitalia were macerated in 10% KOH at room temperature for one day to remove soft tissue, then rinsed in distilled water and dilute acetic acid, and dissected in 80% ethanol. Preparations were then placed into glycerine, with images made with the aid of a digital camera mounted on a stereomicroscope. Genitalia preparations were placed in glycerine in a genitalia vial mounted on the pin beneath the specimen. Terminology follows Winterton (2005) and Winterton and Woodley (2009). In contrast to the scenopinid subfamilies Proratinae and Caenotinae, the male terminalia of Scenopininae are rotated 180°. To avoid confusion with terminology and comparative homology, structures are described and labeled as they are in related flies with terminalia not rotated; therefore the ventral apodeme of the aedeagus described herein is physically located dorsally. The following collection acronyms are cited in the text: California Academy of Sciences, San Francisco, California, USA (CAS),Senckenberg Deutsches Entomologisches Institut, Müncheberg, Germany (DEI), California State Collection of Arthropods, Sacramento, California, USA (CSCA), Bohart Museum of Entomology, University of California, Davis, California, USA (UCDC), the National Museum of Natural History, Smithsonian Institution, Washington, DC, USA (NMNH), and the Natural History Museum, London, United Kingdom (BMNH).Numbers quoted with individual specimens as MEI000000 are unique identifiers in the therevid database MANDALA and are attached to each specimen as a yellow or white label (Kampmeier et al. 2004). Specimen images at different focal points were taken using a digital camera and subsequently combined into a serial montage image using CombineZP. Higher-resolution digital images were also archived in Morphbank with embedded URL links between figure captions and Morphbank images. All new nomenclatural acts and literature are registered in Zoobank as per the recent proposed amendment to the *International Code of Zoological Nomenclature* for a universal register for animal names (Polaszek et al. 2005a,b; Pyle et al. 2008; ICZN 2008).

## Taxonomy

### Heteromphrale

Kröber

Heteromphrale Kröber 1937: 221. – Hardy 1966: 1; Kelsey 1969: 286; 1971: 284; 1973: 332; Woodley 2009: 651. Type species (by original designation): Pseudatrichia chilensisKröber 1928: 31.

#### Diagnosis.

**Figure 1.**
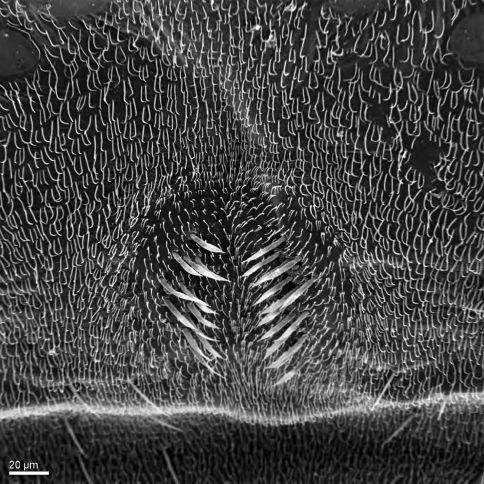
Heteromphrale chilensis (Kröber): Scanning electron micrograph of tergite 2 sensory setal patch.

*Body length:* 2.5–4.0 mm [male], 2.7–5.0 mm [female]. Cream-white with yellowish-brown suffusion; eyes contiguous in male, frons broader than ocellar tubercle in female; antennal flagellum approximately twice length of scape and pedicel combined, attenuate and pyriform, notched apically; scutum light brown to dark grey, with cream-white to yellow patches marginally (pale area often more extensive in female); entire thorax overlain with glaucous pubescence; wing vein M_1_ meeting vein R_5_, forming closed petiolate cell r_5_; R_4_ branching from r_5_ along basal half of cell R_5_; abdomen with tergites either dark brown-grey with pale white to yellow band posteriorly, or vivid white with orange-brown suffusion laterally (and medially in female); tergite 2 sensory setae well defined (Fig. 1); male epandrium split medially as two sclerites, halves sub-quadrangular with posterior margins flared or tapered laterally, or large and globose; epandrium not completely covering gonocoxite ventrally; gonocoxite and aedeagus extended anteriorly from anterior margin of epandrium a relatively short distance; gonocoxite irregular, largely reduced, with strongly sclerotized dorsal process; gonocoxal apodeme relatively thickened; hypandrium as paired lobes, size and shape variable, with margin of setae, but no large setal brushes; lateral aedeagal bulb present; distiphallus bifid, recurved dorsally at base or straight, slender or slightly thickened, arms parallel or divergent. Female sternite 8 longer than tergite 8, almost level with cerci, broadly rounded or weakly emarginate (not forming distinct lobes posterolaterally); 6–7 acanthophorite spines present on lobes of well defined tergite 9+10; furca ring-like, dark-sclerotized; spermathecae paired, sclerotized and irregular-shaped; spermathecal sac simple, minute, elongate.

#### Comments.

**Figure 2.**
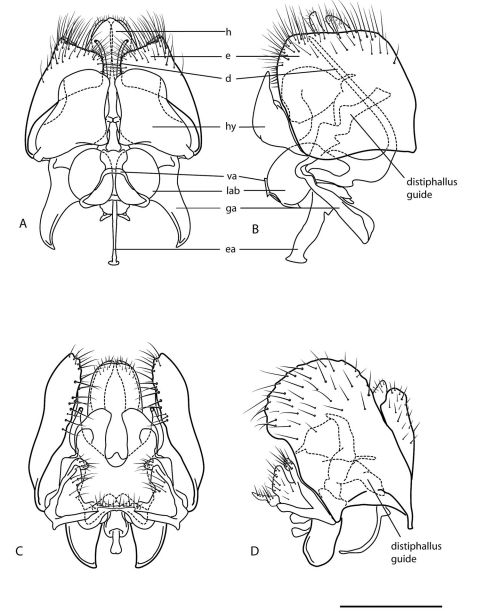
Heteromphrale spp. Male genitalia: **A** Heteromphrale blanca sp. n.: dorsal view **B** same, lateral view **C** Heteromphrale chilensis (Kröber): dorsal view **D** same, lateral view. Scale line = 0.2 mm. Abbreviations: **d** distiphallus **e** epandrium **g** gonocoxite **ga** gonocoxal apodeme **gs** gonostylus **h** hypandrium **hy** hypoproct **lab** lateral aedeagal bulb **va** ventral apodeme of parameral sheath.

**Figure 3.**
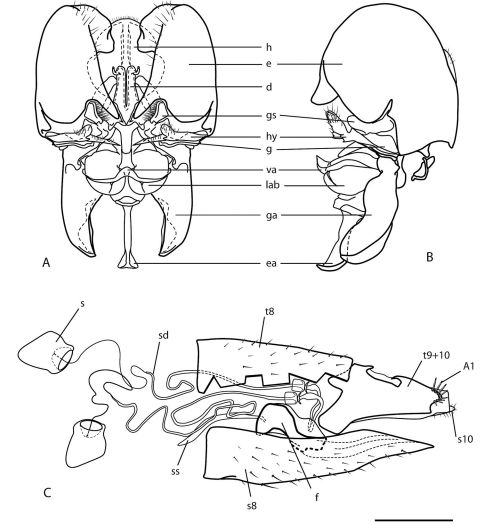
Heteromphrale cyanops (Edwards). Male genitalia: **A** dorsal view **B** same lateral view. Female genitalia: **C** lateral view, with tergite 8 cut away. Scale line = 0.2 mm. Abbreviations: **d** distiphallus **e** epandrium **g** gonocoxite **ga** gonocoxal apodeme **gs** gonostylus **h** hypandrium **hy** hypoproct **lab** lateral aedeagal bulb **va** ventral apodeme of parameral sheath **A1** acanthophorite spines **f** furca **s** spermatheca **sd** spermathecal duct **ss** spermathecal sac **s8** sternite 8 **s10** sternite 10 **t8**tergite 8 **t9+10** tergites 9 and 10.

Heteromphrale is closely related to Brevitrichia, a genus found primarily in western North America and throughout Central America (Kelsey 1969; Woodley 2009). Heteromphrale can be differentiated from Brevitrichia by the shape of sternite 8 in the female (apically emarginate with rounded posterolateral lobes in Brevitrichia), male distiphallus short and thick (relatively long and thread-like in Brevitrichia) and the distiphallus straight (highly reflexed basally in Brevitrichia). The distiphallus of Brevitrichia can be greatly elongated, with the basiphallus reflexed upon itself up to 180° as found in the proratine genus Cyrtosathe Winterton & Metz, 2005 (Winterton and Metz 2005). This complex arrangement of the distiphallus in Brevitrichia often projects anteriorly into the abdominal cavity and is supported by aedeagal guides formed by paired, blade-like extensions of the hypoproct; the aedeagus is largely contained within the genitalic capsule in Heteromphrale, with hypoproct extensions absent. The distinct dorsal processes (physically ventral) on the gonocoxites of Heteromphrale (Figs 2–3) are similar to those found in some species of Propebrevitrichia Kelsey, 1969 (see Winterton 2005) and indicate a likely close relationship between these genera.

#### Distribution.

Southern South America; recorded from Uruguay, Chile and Argentina.

#### Included species.

Heteromphrale blanca sp. n., Heteromphrale chilensis (Kröber) and Heteromphrale cyanops (Edwards).

#### Key to Heteromphrale species

**Table:** 

1	Scutum with glabrous, glossy dorsocentral area (circular in male, linear in female) (Fig. 5C, D); basal antennal flagellomere abruptly pear-shaped; mouthparts tiny, much smaller than oral cavity; female frons with extensive pile (Fig. 4C); abdomen distinctly matte white with brown suffusion laterally and ventrally (Fig. 7A, B), transverse brown line anterior to dark brown spot encompassing tergite 2 sensory patch	Heteromphrale chilensis (Kröber)
–	Scutum with uniform covering of pubescence, lacking a glabrous or glossy mark (Fig. 5A, B, E, F); basal antennal flagellomere more conical-shaped, tapering evenly; mouthparts usually normal-sized, nearly filling oral cavity; female frons with less extensive pile; abdomen in both sexes more extensively dark brown, with white only on posterior margins of tergites (Figs 6A–B, 8A–B)	2
2	Wing with vein R_4_ diverging from vein R_5_ at point in basal quarter of cell R_5_ (Fig. 8B); tergite 2 sensory patch as relatively small single patch, slightly narrowed; male epandrium enlarged, bulbous, without distal fringe of long, white setae on posterior edge (Figs 3A–B, 8A, 10C); female sternite 8 shallowly emarginate posteriorly and without a fringe of long setae; acanthophorite spines stout (Fig. 9G–H)	Heteromphrale cyanops (Edwards)
–	Wing with vein R_4_ diverging from vein R_5_ at point between one-quarter and one-half of cell R_5_ (Figs 6A, B); tergite 2 sensory patch large and distinct, divided into two small patches with setae directed medially; male epandrium not bulbous, size subequal to preceding abdominal segment, with distal fringe of long white setae on posterior edge (Fig. 2A, B, 10A); female sternite 8 distally rounded with dense long thin setae apicolaterally and distally; acanthophorite spines thin and wispy (Fig. 9A–C)	Heteromphrale blanca sp. n.

#### Heteromphrale blanca sp. n.

urn:lsid:zoobank.org:act:4C0F6BCB-3DA3-43E0-BEA8-09C6566B93F9

Figures 2A–B 4A–B 5A–B 6 9A–C 10A 

##### Type material.

###### Holotype

male, “ARGENTINA. La Rioja Prov., Departamento Famatina, 12 km N Pituil, 4,135 ft; 20-X-1997, M.E. Irwin, F.D. Parker, S. Roig, malaise, -28°30'54.36, -67°20'20.04” / “Schlinger Foundation Argentina Expedition, November 1997, ME Irwin, F.D. Parker & S. Roig” / “HOLOTYPUS ♂ Heteromphrale blanca Winterton & Gaimari” [red label]. (MEI165196) (CASC, point mounted, excellent condition).

###### Paratypes.

ARGENTINA: **La Rioja Province:** Departamento Famatina, 12 km N Pituil, 4135 ft. [1260 m], -28°30'54.36, -67°20'20.04, 15.X.1997, M.E. Irwin, F.D. Parker & S. Roig, ex. Malaise trap [1 male (CASC)] (MEI165195); 2 females (CASC) (MEI165197, 165198)]; on leaves of Prosopis tree [1 male (CSCA)]; 53 km from Villa Unión, Route 40, Pedregosa River, 27.XI.1976, ex. sweeping Prosopis chilensis [1 female (USNM)]; Departamento Famatina, 12 km N Pituil, 4135 ft. [1260 m], -28°30'54.36, -67°20'20.04, 15.X.1997, M.E. Irwin, F.D. Parker & S. Roig, ex. Malaise trap [1 male (DEI)] (MEI165199); **Tucuman Province:** 8 km NW Amaichá del Valle, 1847 m, -26°32.35', -65°58.37', 22–25.X.2003, M.E. Irwin & F.D. Parker, ex. Malaise trap in ravine [1 male (CSCA); 1 male (USNM)]. **Salta Province:** 10 km S Cafayete, 26.X–13.XI.2003, M.E. Irwin, F.D. Parker, -26.1514°, -65.9586°, 1644 m, Malaise in Prosopis covered dunes [1 female (CSCA)] (MEI165204).

##### Diagnosis.

Antennal flagellum dark brown to black, conical and evenly tapered distally; mouthparts normal, nearly filling oral cavity; scutum without glabrous dorsocentral patches; wing with vein R_4_ diverging from vein R_5_ at point between one-quarter and one-half of cell R_5_; abdomen dark with pale posterior band on tergites 2–5; tergite 2 sensory patch distinct as two small patches; male epandrium not bulbous, dense fringe of white setae along posterior margin; hypandrium lobes relatively large and sub-triangular; distiphallus with arms parallel; lateral aedeagal bulbs relatively large; female sternite 8 posterior edge rounded; acanthophorite spines elongate and finely tapered, wispy.

##### Description.

**Figure 4.**
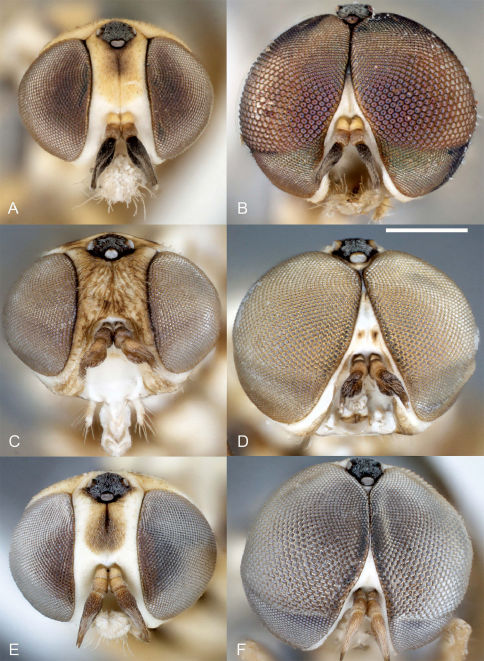
Heteromphrale spp.: Heteromphrale blanca sp. n.: **A** female head, anterior view [Morphbank entry= 579914] **B** male head, anterior view [579921]; Heteromphrale chilensis (Kröber): **C** female head, anterior view [579925] **D** male head, anterior view [579932]; Heteromphrale cyanops (Edwards): **E** female head, anterior view [579935] **F** male head, anterior view [579942]. Scale line = 0.25 mm.

**Figure 5.**
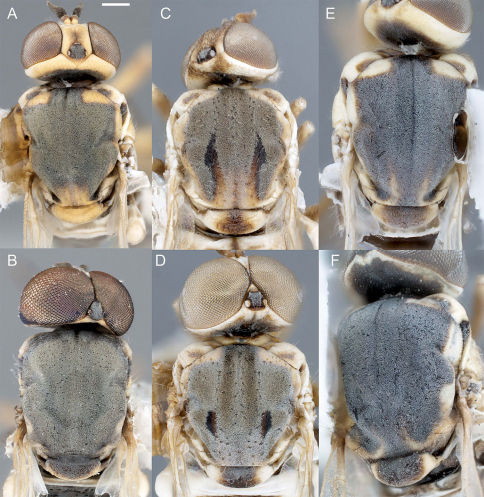
Heteromphrale spp.: Heteromphrale blanca sp. n.: **A** female thorax, dorsal view [579911] **B** male thorax, dorsal view [579919]; Heteromphrale chilensis (Kröber): **C** female thorax, dorsal view [579923] **D** male thorax, dorsal view [579929]; Heteromphrale cyanops (Edwards): **E** female thorax, dorsal view [579933] **F** male thorax, dorsal view [579939]. Scale line = 0.25 mm.

**Figure 6.**
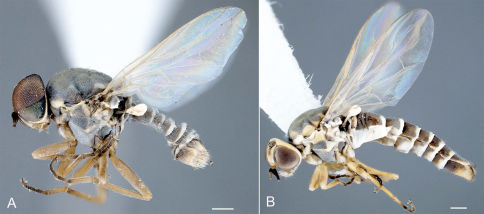
Heteromphrale blanca sp. n.: **A** male, lateral view [579920] **B** female, lateral view [579913]. Scale line = 0.25 mm.

Body length: 2.5–4.0 mm [male], 2.7–4.2 mm [female]. *Head* (Figs 4A–B, 5A–B). Frons, parafacial, face and gena cream-white to yellow; female frons with tan suffusion dorsomedially and surface slightly furrowed medially, sparsely distributed with small, pale setae; male frons with whitish pubescence and dark where eyes are proximate; occiput with yellow suffusion marginally, black medially; face white; flagellum brown to black; scape pale yellow; pedicel yellowish-tan with a few minute pale setae; mouthparts normal-sized, nearly filling oral cavity; pale yellow, including prementum, labellum, labellar setae, and small cylindrical palpus. *Thorax* (Figs 5A–B, 6). Scutum black to grey, small pale yellow areas marginally (postpronotal lobe, notopleuron and supra-alar area), more extensive in female and additionally with yellow on anterior part of scutum adjacent to postpronotum, on postalar callus, and medially on posterior part of scutum; entire thorax overlain with dense glaucous pubescence; scutellum dark medially, yellow marginally (yellow area more extensive in female); scutum without pale setae, some present on postpronotal lobe, anepisternum and katepisternum; prosternum yellow; proepisternum and proepimeron yellow, sometimes with brown on posterior part; anepisternum grey pubescent, yellow in upper part; katepisternum grey pubescent, yellow in posterodorsal corner (in female, along most of dorsal margin); anepimeron grey pubescent anteriorly, yellow posteriorly; meron grey pubescent, except yellow dorsally; coxae light brown to orange; legs tan to dark yellow-orange; distal tarsomeres darker than rest of leg; haltere stem brown, knob white with brown suffusion dorsally; wing venation pale yellow; vein R_4_ diverging from R_5_ at point between one-quarter and one-half of cell R_5_. *Abdomen* (Fig. 6). Segments dark brown-grey with pale white-yellow band posteriorly; sternites dark brown-grey; sparse pale setae on most segments; tergite 2 sensory patch large and distinct, divided medially into two small patches, patch concolorous with rest of tergite. *Male genitalia* (Figs 2A–B, 10A). Epandrium sub-quadrangular, outer margins tapered, dark brown in basal half and pale yellow in distal half, white setal fringe along margin; hypandrium lobes relatively large, triangular; gonocoxite with darkly sclerotized, narrowly acuminate, dorsal process; gonostylus complex, apparently fused to gonocoxites, with posteriorly directed margin fringed with setae, and hook-like process dorsally, inner lobe triangular and fused medially; gonocoxal apodeme broadly flattened, curved medially; ejaculatory apodeme relatively elongate, directed anteriorly; lateral aedeagal bulbs large, round; distiphallus elongate, arms proximate and parallel, broadly curved ventrally at base then straight along distal length. *Female genitalia* (Fig. 9A–C). Sternite 8 with posterior edge rounded, with posterolateral part fringed with long wispy setae; acanthophorite spines elongate and curved, becoming wispy and hair-like.

##### Comments.

Heteromphrale blanca sp. n. is very similar to Heteromphrale cyanops in overall body color, but differs considerably in male genitalic morphology and in the shape of the female sternite 8. As in Heteromphrale chilensis, the tergite 2 sensory patch is large and distinct, and is divided into two small patches with the setae directed medially. In males, the non-bulbous epandrium distinguishes this species and Heteromphrale chilensis from Heteromphrale cyanops, but the dense fringe of white setae along the posterior margin, distinguishes Heteromphrale blanca sp. n. from Heteromphrale chilensis. In females, the rounded sternite 8 distinguishes Heteromphrale blanca sp. n. from Heteromphrale cyanops, and the fringe of long wispy setae and elongate wispy acanthophorite spines distinguishes this species from Heteromphrale cyanops and Heteromphrale chilensis.

##### Distribution.

Known only from Argentina (La Rioja, Salta and Tucuman Provinces).

##### Etymology.

The specific epithet is a Latin adjective – *blanca* – meaning white, referring to the dense fringe of white setae along the posterior margin of the epandrium.

#### Heteromphrale chilensis

(Kröber)

Figures 1 2C–D 4C–D 5C–D 7 9C–E 10B

Pseudatrichia chilensis Kröber 1928: 31.
Heteromphrale chilensis  (Kröber). – Kröber 1937: 221; Hardy 1966: 2; Kelsey 1969: 286; 1971: 284.

##### Type material.

###### Holotype

female, label data: “CHILE Concepción, P. Herbst” / “coll. Lichtwardt” / “Pseudatrichia chilensis det. Kröber” / “HOLOTYPUS”. (DEI) **(**micropinmounted**,** reasonable condition except abdomen greasy and antennae missing).

##### Other material examined.

CHILE: **Elqui Province:** 10 km N La Serena, 10 m, -29°49.27', -71°16.20', 8.X.2003, M.E. Irwin, ex. hand net in coastal dunes [3 males (CASC) (MEI165208, 165209, 165210), 1 female (CASC) (MEI165211), 3 males (CSCA) (MEI165206, 165207 165212)].

##### Diagnosis.

Antennal flagellum orange brown, abruptly pear-shaped; female frons with extensive pile; mouthparts much smaller than oral cavity; scutum with glabrous, glossy dorsocentral spot (linear in female); wing with vein R_4_ diverging from R_5_ at point in basal quarter of cell r_5_; abdomen vivid matte-white with brown suffusion laterally (also medially in female), and with dark brown spot encompassing tergite 2 sensory patch; tergite 2 sensory patch distinct as two small patches; male epandrium not bulbous, without dense fringe of setae; hypandrium lobes sub-triangular with sclerotized lateral margins; distiphallus arms divergent; lateral aedeagal bulbs relatively small; female sternite 8 rounded posteriorly, without fringing elongate setae; acanthophorite spines robust and stout.

**Figure 7.**
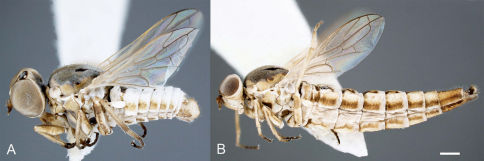
Heteromphrale chilensis (Kröber): **A** male, lateral view [579931] **B** female, lateral view [579924]. Scale line = 0.25 mm.

##### Redescription.

Body length: 2.6–3.2 mm [male], 4.8 mm [female]. *Head* (Figs 4C–D, 5C–D). Frons cream-white, female frons with yellow to light brown patch dorsomedially, sometimes more extensive brown-orange suffusion, surface wrinkled, sparsely distributed with small, pale setae; parafacial in male dark orange medially, white along eye margin (in female entirely yellow); ocellar triangle grey pubescent, raised, with anterior ocellus slightly larger than posterior ocellus; occiput and postgena dark with pale yellow with orange-brown suffusion marginally; face white with brown suffusion; mouthparts pale, relatively small in size, much smaller than oral cavity; pale yellow, including labellum, labellar setae, and small cylindrical palpus; prementum in male dark orange (in female pale yellow); flagellum orange-brown, abruptly pyriform, tapered distally; scape and pedicel brown with a few minute pale setae. *Thorax* (Figs 5C–D, 7). Scutum light brown to grey, yellow on postpronotal lobe, anterior part of scutum adjacent to postpronotum, notopleuron, supra-alar area and postalar callus (in female, with yellow more extensive in these areas); entire thorax overlain with dense glaucous pubescence; glossy black dorsocentral patches present at point posterior third of scutum, patches round in male, elongate in female, paired median brown vittae in anterior half; scutellum dark medially, pale marginally; prosternum yellow, bare; proepisternum and proepimeron orange (in female yellow); anepisternum orange in dorsal half and along posterior margin (in female yellow), except white along dorsal margin and grey to brown pubescent anteroventrally; katepisternum grey to brown pubescent, except orange in upper part (in female yellow); anepimeron white to yellow, darkened anteriorly; meron shining glossy brown, except white pubescent dorsally; legs pale cream with uniform or mottled brown suffusion, tibiae orange, with dorsal surface pale; hind tibia becoming darker distally; tarsi dark. *Wing*. Venation pale brown; vein R_4_ diverging from R_5_ at point in basal quarter of cell r_5_; aberrant specimens with either spurious vein present between distal part of R_5_ and C (Fig. 7B) or R_4_ incomplete basally; haltere mostly cream-white. *Abdomen* (Fig. 7). Vivid matte-white to cream with brown suffusion laterally; dark brown band posteriorly (more obvious in female), tergite 2 with dark brown band level with and encompassing sensory setal patch; sternites white with brown suffusion laterally; sparse elongate setae on most segments. *Male genitalia* (Figs 2C–D, 10B). Epandrium brown basally and pale yellow marginally; sub-quadrangular with posterior margins tapered, fine setae along margin; hypandrium halves small, sub-triangular with multiple lobes directed posteromedially, dark sclerotized along anterior and lateral margins; gonocoxite with darkly sclerotized, acuminate, dorsal process; gonostylus large, posteriorly directed and united medially, apparently fused to gonocoxites; gonocoxal apodeme broadly flattened, outer margin curved, inner margin straight; ejaculatory apodeme minute, directed ventrally; lateral aedeagal bulbs small; ventral apodeme dark sclerotized; distiphallus divergent laterally around gonostylus, medially directed process from between distiphallus projecting towards hypoproct, curved anteriorly, apex spatulate. *Female genitalia* (Figs 9C–E). Sternite 8 with posterior edge rounded, with longish setae around fringe; acanthophorite spines long and robust.

##### Comments.

Originally described in Pseudatrichia, Kröber (1937) later erected Heteromphrale to accommodate this species. Although the female holotype is faded, encrusted with naphthalene and dust, and the abdomen is greasy, the distinctive bone-white color of the abdomen is still observable, along with the brown coloration around the tergite 2 sensory patch. In the original description, Kröber (1928) described the flagellum as red-yellowish color, but the antennae are now lost from the type specimen.

Heteromphrale chilensis is easily distinguished from other species of Heteromphrale by the glabrous dorsocentral patches on the scutum (elongate in females), mostly bone-white coloured abdomen, basally bulbous antennal first flagellomere, and the relatively tiny mouthparts. As in Heteromphrale blanca sp. n., the tergite 2 sensory patch is large and distinct, and is divided into two small patches with the setae directed medially, and the epandrium is not bulbous as in Heteromphrale cyanops, but in Heteromphrale chilensis there is no dense fringe of long white setae. Also like Heteromphrale blanca sp. n., the female sternite 8 is rounded, but in Heteromphrale chilensis, the edge is not fringed with long wispy setae, and the acanthophorite spines are robust.

##### Distribution.

Known from Chile (Biobío Region (Concepcíon Province) and Coquimbo Region (Elqui Province)).

#### Heteromphrale cyanops

(Edwards)

Figures 3 4E–F 5E–F 8 9G–I 10C

Pseudomphrale cyanops Edwards 1932: 259. – Kröber 1937: 212; Hardy 1966: 2.
Heteromphrale cyanops  (Edwards) – Kelsey 1969: 286.

##### Type material.

###### Holotype

male, URUGUAY: Montevideo, 21.i.1927, F. & M. Edwards (MEI165200). (BMNH) (excellent condition).

##### Other material examined.

ARGENTINA: **Catamarca Province:** 50 km W Andalgala, 31.X.1972, G.E. Bohart [1 male (UCDC)]; Andalgala, 4.XI.1972, G.E. Bohart, Prosopis alba [1 male (UCDC)]; 28 km SE Tinogasta, 1100 m, -28.2450°, -67.4557°, 17.X.1997, M.E. Irwin, F.D. Parker & S. Roig, ex. inland dunes [4 males (CSCA)]. **La Rioja Province:** Departamento Famatina, 12 km N Pituil, 4135 ft. [1260 m], -28°30'54.36, -67°20'20.04, 15.X.1997, M.E. Irwin, F.D. Parker & S. Roig, ex. Malaise trap [1 female (CASC), 1 female (CSCA)], ex. leaves of Prosopis tree [1 male (CASC)], 20.X.1997, ex. leaves of Prosopis tree [1 female (CASC)]. 16 km NE Pagancillo, Route 18, 28.XI.1976, ex. sweeping Prosopis chilensis [2 males (USNM)]. **Mendoza Province:** Departmento de Levalle, 20 km N Parque Telteca, -32.2916°, -67.3878°, 10.x.1997, M.E. Irwin, F.D. Parker, S. Roig, ex. Cerecidium blooms in sandy area [1 male (CASC)]. **Salta Province:** 10 km S Cafayete, 1644 m, -26°09.05', -65°57.31', 22–26.X.2003, M.E. Irwin & F.D. Parker, ex. Malaise trap in Prosopis-covered dunes [1 female (CASC), 1 female (CSCA)]. **Tucuman Province:** 8 km NW Amaichá del Valle, 1847 m, -26°32.35', -65°58.37', 22–25.X.2003, M.E. Irwin & F.D. Parker, ex. Malaise trap in ravine [1 female (CASC)].

##### Diagnosis.

Antennal flagellum brown, conical and evenly tapered distally; mouthparts normal, nearly filling oral cavity; scutum without glabrous dorsocentral patches; wing with vein R_4_ diverging from vein R_5_ at point in basal quarter of cell r_5_; abdomen dark with pale posterior band on tergites 2–5; tergite 2 sensory patch relatively small, as single narrowed patch; male epandrium relatively large and bulbous, without distinctive white setal fringe along posterior margin; hypandrium small, lobes irregular; distiphallus arms parallel; lateral aedeagal bulb relatively large; female sternite 8 shallowly emarginate posteriorly, without fringing elongate setae; acanthophorite spines short and stout.

**Figure 8.**
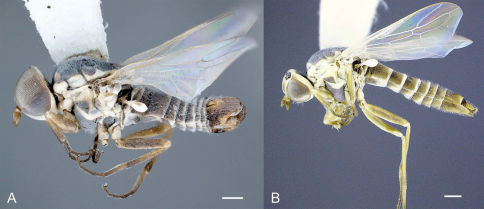
Heteromphrale cyanops (Edwards): **A** male, lateral view [579941] **B** female, lateral view [579934]. Scale line = 0.25 mm.

##### Redescription.

**Figure 9.**
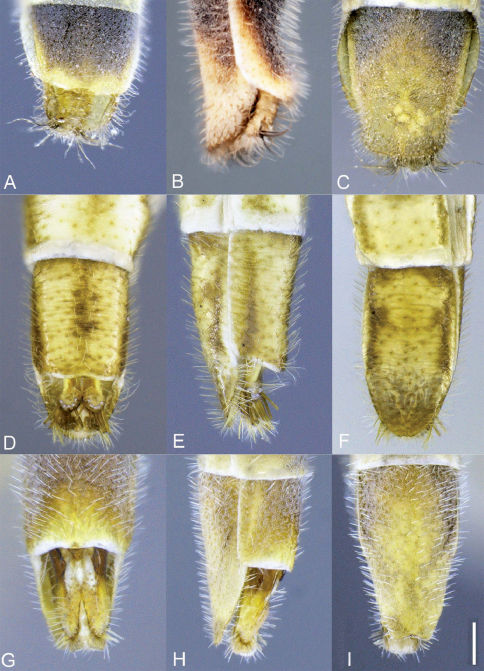
Heteromphrale spp., female terminalia: Heteromphrale blanca sp. n.: **A** dorsal view [579915] **B** lateral view [579916] **C** ventral view [579917]; Heteromphrale chilensis (Kröber): **D** dorsal view [579926] **E** lateral view [579927] **F** ventral view [579928]; Heteromphrale cyanops (Edwards): **G** dorsal view [579936] **H** lateral view [579937] **I** ventral view [579938]. Scale line = 0.25 mm.

**Figure 10.**
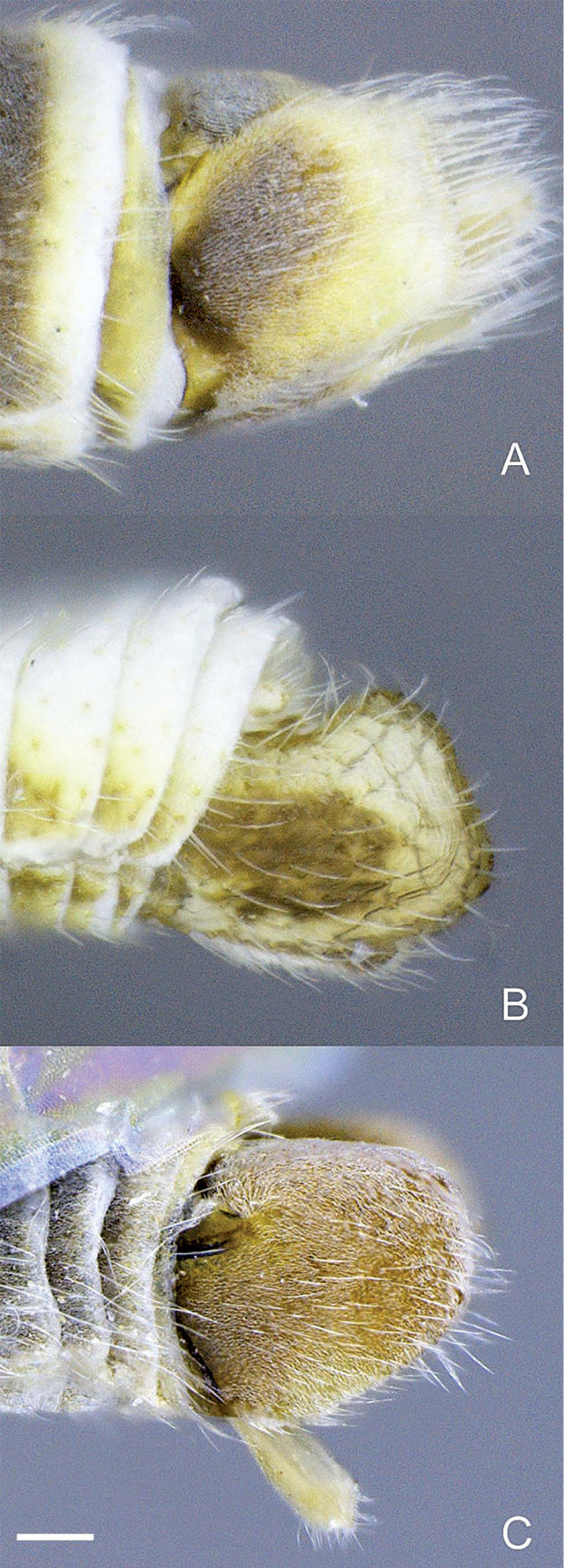
Heteromphrale spp., male terminalia: **A** Heteromphrale blanca sp. n., lateral view [579922] **B** Heteromphrale chilensis (Kröber), lateral view [579930] **C** Heteromphrale cyanops (Edwards), lateral view [579940]. Scale line = 0.25 mm.

Body length: 2.5–3.5 mm [male], 3.0–4.0 mm [female]. *Head* (Figs 4E–F, 5E–F). Frons, parafacial, face and gena cream-white to yellow (in female, with tan suffusion on frons, with median furrow), sparsely distributed with small, pale setae; ocellar triangle grey pubescent, slightly raised, with anterior ocellus slightly larger than posterior ocellus occiput and postgena dark, pale marginally with yellow suffusion; face white; mouthparts pale yellow, including labellum, labellar setae, and small cylindrical palpus; prementum pale yellow to slightly darker yellow; antennae with flagellum brown, evenly tapered; scape and pedicel pale brown-orange with a few minute pale setae. *Thorax* (Figs 5E–F, 8). Scutum black to grey, cream-white to yellow areas on postpronotal lobe, anterior part of scutum adjacent to postpronotum, notopleuron, supra-alar area and postalar callus with slightly darker, thin, median stripe (more extensive in female in these areas, and additionally with yellow medially on posterior part of scutum, and a slight medial brownish mark extending from scutellum); thorax mostly overlain with dense glaucous pubescence; scutum largely without setae, some present on postpronotal lobe, anepisternum and katepisternum; scutellum dark medially, pale marginally; prosternum yellow, bare; proepisternum and proepimeron yellow, sometimes slightly darker on posterior part; anepisternum yellow in dorsal half and along posterior margin, grey pubescent anteroventrally; katepisternum grey pubescent, except yellow along entire dorsal margin; anepimeron yellow, usually with some grey pubescence anteriorly; meron grey pubescent, except yellow dorsally; coxae light brown; legs pale cream with uniform brown suffusion; basitarsi orange with slightly darker distal tip, and remaining tarsomeres dark orange. *Wing*. Venation pale yellow; vein R_4_ diverging from R_5_ at point in basal quarter of r_5_; haltere with stem mostly brown, distal part of stem and entire knob white. *Abdomen* (Fig. 8). Tergites dark brown pubescence with pale white band posteriorly; with pale setae, longer laterally; tergite 2 sensory patch small and inconspicuous, as single small, brown narrow patch, completely encompassed within dark brown pubescence; sternites white posteriorly, dark brown anteriorly and laterally; with sparse short pale setae. *Male genitalia* (Figs  3, 10C). Epandrium robust, globose, dark orange, with sparse, small white setae; hypandrium lobes small, membranous, sub-triangular, each with two lobes directed posteromedially; gonocoxite with large, darkly sclerotized, anvil-like dorsal process; gonostylus complex, apparently fused to gonocoxites, outer lobe L-shaped with posteriorly directed margin fringed with setae, inner lobe triangular and fused medially; gonocoxal apodeme broadly flattened, and curved medially; ejaculatory apodeme relatively elongate, directed anteriorly; lateral aedeagal bulbs large; ventral apodeme dark sclerotized; distiphallus parallel, ventrally directed towards apex. *Female genitalia* (Fig. 9G–I). Sternite 8 with posterior edge weakly emarginate, margin thin; acanthophorite spines stout.

##### Comments.

Heteromphrale cyanops was originally described in the genus Pseudomphrale by Edwards (1932) based on a single male specimen from Uruguay. Edwards (1932) noted that the specimen was taken in a sandy spot near the shore, and also noted that the eye in life was deep blue in color; this eye color is retained in some specimens. Kelsey (1969) subsequently transferred the species to Heteromphrale. Heteromphrale cyanops is very similar to Heteromphrale blanca sp. n. in overall body coloration, but is easily distinguished from both other species of Heteromphrale by the greatly enlarged, bulbous epandrium in the male, the shallowly emarginate posterior edge of sternite 8 in the female, and by the tergite 2 sensory patch being a relatively small, singular, narrowed patch.

##### Distribution.

Known from Argentina (Catamarca, La Rioja, Mendoza, Salta and Tucuman Provinces) and Uruguay (Montevideo Department).

## Supplementary Material

XML Treatment for Heteromphrale
